# Retraction: Tetrahydrocurcumin induces mesenchymalepithelial transition and suppresses angiogenesis by targeting HIF1α and autophagy in human osteosarcoma

**DOI:** 10.18632/oncotarget.28888

**Published:** 2026-05-20

**Authors:** Yan Zhang, Ying Liu, Jilong Zou, Lixin Yan, Wei Du, Yafeng Zhang, Hanliang Sun, Peng Lu, Shuo Geng, Rui Gu, Hongyue Zhang, Zhenggang Bi

**Affiliations:** ^1^Department of Orthopaedics, The First Affiliated Hospital of Harbin Medical University, Nangang District, Harbin, P.R. China; ^2^Department of Pharmacy, Harbin Medical University, Harbin, Heilongjiang, P.R. China; ^3^Department of Biopharmaceutical Sciences, College of Pharmacy, Harbin Medical University-Daqing, Daqing, Heilongjiang, P.R. China; ^4^School of Pharmacy, Harbin University of Commerce, Harbin, Heilongjiang, P.R. China; ^5^Department of Orthopaedics, Baoquanling Central Hospital, Baoquanling, Heilongjiang, P.R. China

**This article has been retracted:** Oncotarget has concluded its investigation of this paper, which identified the following issues:

- Figure 1D: Overlap between three wound healing assay images at the 0 timepoint.
- Figure 1E: Overlap between THC 12.5 uM and THC 25 uM panels in the U-2OS Migration row; overlap between THC 12.5 uM and THC 25 uM panels in the SaOS-2 Migration row; and overlap between the Control and THC 12.5 uM panels in the SaOS-2 Invasion row. Additionally, the Control panel in the Invasion row is duplicated from a concurrently published article by the same group [1].
- Figure 3A: The western blot for MG-63/HIF-1a is a duplicate of the Figure 4E MG-63/NF-kB/P65 blot; the U-2OS/HIF-1a blot is a duplicate of the Figure 4E U-2OS/NF-kB/P65 blot.
- Figure 3F: The last three bands of the western blot for U-2OS/Snail duplicate the first three bands of Figure 5D, U-2OS/VEGF.
- Figure 4A, 4D: The U-2OS/b-actin (Figure 4A) and MG-63/b-actin (Figure 4D) images are duplicates.
- Figure 4E: The western blots for MG-63/HSP-90 and U-2OS/HSP-90 are duplicates.

The authors did not respond to requests for comment or provide the original data. Consequently, the editorial decision has been made to retract this paper. While Oncotarget has reached out to all authors to notify them of this action, no response has been received.

Original article: Oncotarget. 2017; 8:91134–91149. 91134-91149. https://doi.org/10.18632/oncotarget.19845
